# Improved Transformation and Regeneration of *Indica* Rice: Disruption of *SUB1A* as a Test Case via CRISPR-Cas9

**DOI:** 10.3390/ijms22136989

**Published:** 2021-06-29

**Authors:** Yuya Liang, Sudip Biswas, Backki Kim, Julia Bailey-Serres, Endang M. Septiningsih

**Affiliations:** 1 Department of Soil and Crop Sciences, Texas A&M University, College Station, TX 77843, USA; liangy27@msu.edu (Y.L.); sudipbmb@tamu.edu (S.B.); uptfamily@snu.ac.kr (B.K.); 2 Department of Plant Biology, Michigan State University, East Lansing, MI 48823, USA; 3 Department of Agriculture, Forestry and Bioresources, Research Institute for Agriculture and Life Sciences and Plant Genomics and Breeding Institute, Seoul National University, Seoul 08826, Korea; 4 Center for Plant Cell Biology, Botany and Plant Sciences, University of California, Riverside, CA 92521, USA; serres@ucr.edu

**Keywords:** gene editing, CRISPR-Cas9, rice, *indica*, *SUB1A*, tissue culture

## Abstract

Gene editing by use of clustered regularly interspaced short palindromic repeats (CRISPR) has become a powerful tool for crop improvement. However, a common bottleneck in the application of this approach to grain crops, including rice (*Oryza sativa*), is efficient vector delivery and calli regeneration, which can be hampered by genotype-dependent requirements for plant regeneration. Here, methods for *Agrobacterium*-mediated and biolistic transformation and regeneration of *indica* rice were optimized using CRISPR-Cas9 gene-editing of the submergence tolerance regulator *SUBMERGENCE 1A-1* gene of the cultivar Ciherang-Sub1. Callus induction and plantlet regeneration methods were optimized for embryogenic calli derived from immature embryos and mature seed-derived calli. Optimized regeneration (95%) and maximal editing efficiency (100%) were obtained from the immature embryo-derived calli. Phenotyping of T_1_ seeds derived from the edited T_0_ plants under submergence stress demonstrated inferior phenotype compared to their controls, which phenotypically validates the disruption of *SUB1A-1* function. The methods pave the way for rapid CRISPR-Cas9 gene editing of recalcitrant *indica* rice cultivars.

## 1. Introduction

The clustered regularly interspaced short palindromic repeats-Cas9 nuclease (CRISPR-Cas9) technology for genome editing has become a powerful tool for gene-targeted crop improvement. This technique has been applied to various crops, including cereals, such as wheat [1,2,3], corn [4,5,6], and rice [7,8,9]. The CRISPR-Cas9 reagents, including the promoter-guide (g)RNA and promoter-Cas9 gene constructs in a single vector [10], are delivered into the host plant where the gRNA-Cas9 complex guides the position of Cas9 protein cleavage in the host genome through non-homologous end-joining (NHEJ) or homologous recombination (HR) [11]. *Agrobacterium*-mediated transformation via T-DNA insertion is the most common and stable method to deliver transgene vectors into a host plant [12].

In rice (*Oryza sativa* L.), transformation protocols of *japonica* cultivars using mature seeds as explants have long been established [13]. Due to poor callus induction and regeneration and low transformation efficiency of seed explants, *indica* cultivars are more challenging to transform [14,15,16]. The use of immature embryos as explants is a viable alternative, but time and labor are needed to dissect the immature embryos. Hiei and Komari’s protocol [15] requires 74 days, not including growing plants for the immature embryos, to generate the transgenic plants with up to 13% transformation efficiency. Several studies have reported efficient *indica* rice transformation protocols using mature seeds [17,18,19], but these are largely genotype specific [20,21]. For example, Sahoo’s protocol [19] suggested that the transgenic plants derived from mature seeds of IR64, PB1, CSR10, and Swarna can be obtained in 65 days with up to 46% transformation efficiency and up to 92% regeneration frequency for the untransformed calli and up to 59% for the transformed calli. Meanwhile, Sahoo and Tuteja’s protocol [22] suggested that the transgenic plants derived from mature seeds of IR64 can be obtained in 64 days with 12% transformation efficiency.

Ciherang-Sub1 is an elite *indica* rice cultivar derived from Ciherang, a popular variety from Indonesia [23]. This submergence tolerant variety has been released in several countries in Southeast and South Asia and used as a new elite parent for further rice improvement [24,25,26,27,28]. Ciherang-Sub1 explants are recalcitrant in shoot regeneration, hampering its improvement via transgene introduction or genome editing. The aim of this study was to optimize for *Agrobacterium*-mediated transformation and regeneration methods for *indica* rice from immature embryos as explants. Additionally, we also wanted to compare it with particle bombardment-mediated transformation and regeneration using mature seed-derived calli to avoid having to grow out plants and dissect immature embryos after flowering. By use of Ciherang-Sub1 as a target for gene editing, we demonstrate that disruption of *SUB1A-1* alone is sufficient to limit key traits associated with the quiescence strategy of submergence survival [29,30,31,32].

## 2. Results and Discussion

### 2.1. Optimization of Callus Induction Medium for Mature Seeds

To optimize callus induction medium (CIM) for mature seeds, several medium components were modified from the previous *japonica* and *indica* rice tissue culture protocols [15,19]. First, we compared five different auxin to cytokinin compositions of callus induction formulae (Table A1) as follows: (1) with 3 mg 2,4-Dichlorophenoxyacetic acid (2,4-D) and 0.25 mg 6-Benzylaminopurine (6BA) [19]; (2) 2.5 mg 2,4-D and 0.15 mg 6BA [22]; (3) 3 mg 2,4-D and 0.2 mg 6BA; (4) 3 mg 2,4-D and 0.2 mg 6BA with phytagel increased to 4 g/L; and (5) 3 mg 2,4-D and 0.3 mg 6BA. Calli induced from Formula (1) tended to be darker (Figure S1A). Calli induced from Formulae (1) and (2) were smaller (Figure S1) than Formula (5) (Figure S2). However, calli induction using Formulae (3) and (4) had the smallest calli. Hence, increasing phytagel from 3 g/L to 4 g/L did not show any significant difference between Formulae (3) and (4). This experiment suggests that medium Formula (5) with 3 mg 2,4-D and 0.3 mg 6BA had the best callus induction rate and quality for Ciherang-Sub1.

**Figure 1:**
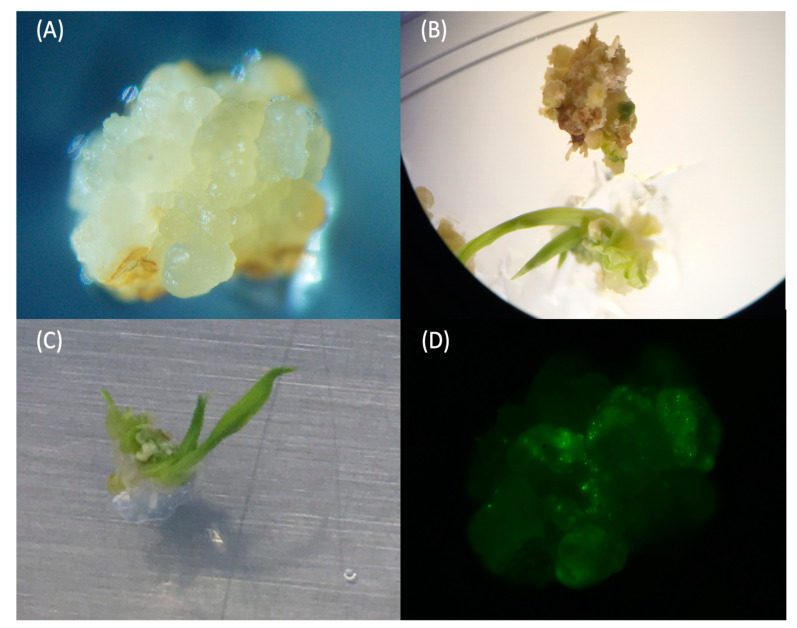
Calli’s performances under a microscope. (**A**) The optimal callus for bombardment. (**B**) Shoot formation of callus on shoot induction medium (SIM). (**C**) Leaf formation of callus on shoot induction medium (SIM). (**D**) YFP fluorescence detection in callus five days after biolistic bombardment pPTN-EYFP vector.

When seeds were cultured on CIM with an optimal auxin to cytokinin ratio (Formula (5)), nearly all the seeds could be induced for embryogenic calli (Figure S2). After 10–14 days of culturing, roots were removed, and calli were cut into 3 to 4 smaller pieces and sub-cultured for another 14–18 days. These divided-calli could facilitate callus propagation and be beneficial for the transformation process. More than 80% of seeds had propagated new calli after 16 days of sub-culturing in fresh CIM (Figure S2B). The optimal callus for transformation should have a creamy or yellow appearance, with a visible differentiated shape on the surface (Figure 1A). It also should not be too wet, such as a juicy surface or visible water drop on the surface, or too fragile, which could be easily crushed by tweezers.

Previous studies had revealed that not only different plant hormone ratios but also variation in carbon sources could have significant effects on tissue culture [33]. In our study with Ciherang-Sub1, maltose was the optimal carbon source for the callus induction stage. We observed that calli turned brown and dry when sucrose was used instead of maltose. In this case, sucrose may cause greater osmotic potential than maltose, and it also has a different organogenesis rate [34]. Previous studies also showed that sucrose replacement with maltose could improve callus induction for spring wheat (*Triticum aestivum* L.) and some *indica* rice varieties [19,35]. However, for the callus induction in *japonica* rice cultivars, sucrose is used as the carbon source [15].

In organogenesis, auxin and cytokinin are the major phytohormones, which control the different organogenesis stages. For example, auxin to cytokinin ratio equal to 3:0.2 will induce callus, 0.03:1 can induce shoot regeneration, and 3.0:0.02 can induce roots [36]. Compared to other *indica* cultivars IR64 and IR64-Sub1, Ciherang-Sub1 has slower callus formation and propagation stages. Therefore, we slightly adjusted the concentration of 2,4-D, a synthetic auxin, and 6BA, a cytokinin source, from two published protocols [19,22]. With a higher 2,4-D supply, the callus became smaller, and more lateral roots differentiated from mature seeds’ main roots. On the other hand, when we slightly increased 6BA concentration from 0.25 mg/L to 0.3 mg/L, the callus formation and propagation became more vigorous. Therefore, we recommend an auxin to cytokinin ratio of 3:0.3 as the optimal ratio for callus induction in Ciherang-Sub1. Our study also suggests that callus with ideal shape developed after 30 days of callus induction is an optimal target for biolistic bombardment. Overall, this particle bombardment protocol using mature seeds as explant requires 80 days from callus induction to the transfer of a transgenic T_0_ seedling to soil (Figure S3).

### 2.2. Optimization of Regeneration Medium for Mature Seeds

For the testing of the regeneration medium for shooting, three different cytokinin to auxin ratios were tested (Table A2), including (1) 2 mg kinetin and 0.2 mg 1-Naphthaleneacetic acid (NAA) [19]; (2) 3 mg kinetin and 0.1 mg NAA; and (3) 3 mg kinetin and 0.1 mg NAA with agarose reduced from 8 g/L to 7 g/L (see Method section). The optimal actively dividing calli were selected under a microscope and transferred onto three regeneration media. In the testing phase and the evaluation of regeneration efficiency, all three media had no antibiotics added. The developing and testing processes were conducted using calli derived from mature seeds.

With 2 mg kinetin and 0.2 mg NAA medium (Formula (1)), green tissues could be observed around ten days after transferring onto the regeneration medium; however, around 15% of calli became dry and brown after turning green. In contrast, for 3 mg kinetin and 0.1 mg NAA formula with reduced agarose (Formula (3)), the light green tissue formation could be observed as early as five days after being transferred onto the regeneration medium. After being transferred onto the optimized shoot induction medium (SIM), the yellow or creamy color callus would partially turn light green from the corner. Subsequently, the green became darker, a part expanded, and shoots began to appear. The spikes would elongate and differentiate into leaves (Figure 1A–C). This type of callus was tested for the pPTN-EYFP expression vector beforehand to ensure that we had suitable calli to work with (Figure 1D). In total, we observed 19 out of 20 calli were regenerated after 5–14 days, being transferred onto optimized SIM without antibiotic (Figure S4A, B), indicating that we had a 95% regeneration rate of Ciherang-Sub1. This optimal regeneration medium was also tested for IR64-Sub1 mature seeds, and it worked well, showing its potential to be used for other *indica* varieties (Figure S4C).

We observed that calli had a similar response to two media (Formulae (2) and (3)) with the same cytokinin to auxin ratio but with different agarose concentrations. Notably, the lower agarose concentration medium allowed calli to turn green 2–3 days earlier and reduce brown calli formation. Hence, the regeneration medium containing 3 mg kinetin and 0.1 mg NAA with 7 g agarose to solidify had the highest percentage of green callus and had faster progress. These results are consistent with previous studies, which revealed that a low auxin:cytokinin ratio generally promotes shoot formation from callus [36,37,38]. We also noticed that reduced agarose concentration from 8 g/L to 7 g/L expedites the regeneration of Ciherang-Sub1.

Many rice tissue culture studies apply fungicide (Benomyl) on the roots or in a hydroponic solution after plants are moved out from the rooting medium [39,40,41]. However, our study used a more convenient protocol where fresh tap water was adequate for rice acclimation. The new roots could be observed 5–7 days after culturing in water (Figure S4D). This is the sign that the plant is ready to be transferred to soil. After moving to the soil, the regenerated plants can be treated as normal plants. No extra cover is needed for maintaining the humidity. Instead, air temperature and aeration are critical points. Air temperature that is too high (>30 °C) or poor aeration will cause plants to grow poorly and eventually die.

### 2.3. Regeneration Performance of Immature Embryo from Agrobacterium-Mediated Transformations Using the Optimized Shoot Induction Medium

**Figure 2:**
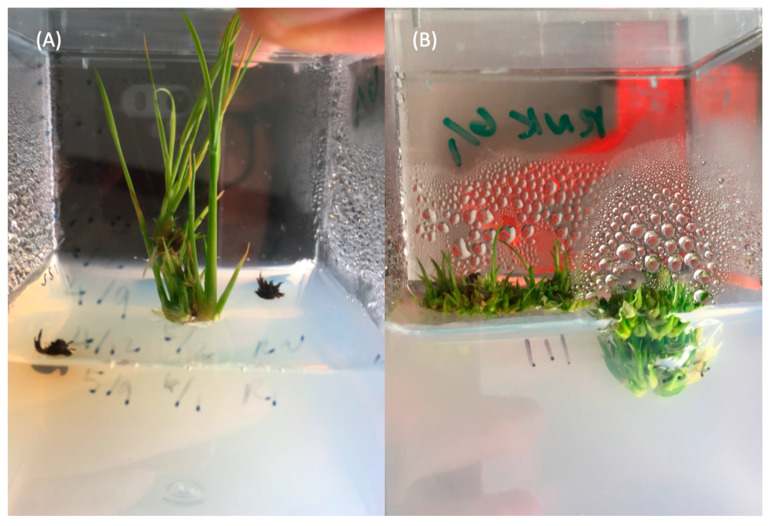
Performance of Ciherang-Sub1 calli in regeneration media. (**A**) Calli in the SIM medium (optimized medium); and (**B**) calli in regeneration medium for type 4 *indica* varieties [15].

In the first batch of immature embryo transformation, a set of calli were transferred onto the optimized SIM, and another set of calli were transferred onto Hiei and Komari regeneration medium [15]. The results suggested that the optimized SIM works very well for calli induced from the immature embryo (Figure 2A). In contrast, we found that calli on the Hiei and Komari regeneration medium [15] type 4 *indica* variety regeneration medium tended to generate many tillers from the bottom instead of having normal shoot elongation (Figure 2B). Despite the high genetic background similarity between Ciherang-Sub1 with IR64-Sub1 and IR64, which belong to the type 4 *indica* variety [15], the regeneration medium for type 4 *indica* cultivar, IR64, was not suitable for Ciherang-Sub1, presumably due to genotype-dependent specificity in the regeneration protocol.

### 2.4. Validation of Transgenic Plants Derived from Immature Embryos

**Figure 3:**
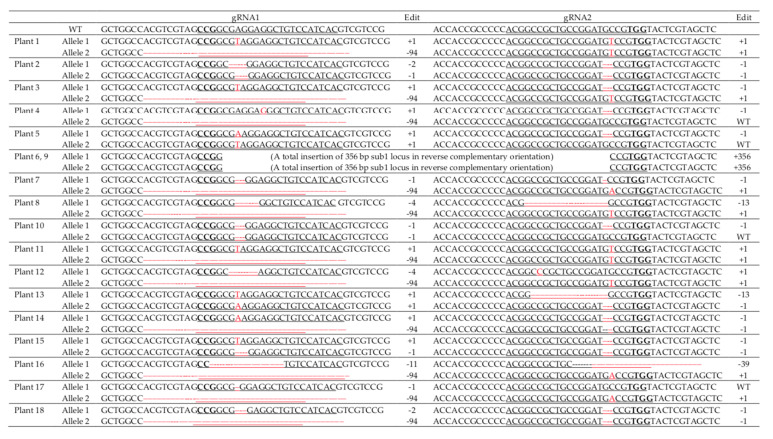
Sanger sequencing results of transgenic plants derived from immature embryo transformation. Two gRNAs are underlined. PAM sequences are highlighted in bold. gRNA1: 5′-**CCG**GCGAGGAGGCTGTCCATCAC-3′; gRNA2: 5′-ACGGCCGCTGCCGGATGCCG**TGG**-3′. gRNA1 and gRNA2 are 350 bp apart. "WT" indicates that the allele was not edited (wild type).

A total of 18 transgenic plants were obtained using immature embryo transformation generated from the optimized regeneration medium. The transgenic plants first were screened by the Cas9 specific primers. Genotyping by PCR confirmed that all 18 hygromycin-resistant transgenics (T_0_) were positive for Cas9 (Figure S5). Sanger sequencing confirmed edits in the regions of *SUB1A* targeted by the two gRNAs in all transgenic plants, with biallelic mutations in most cases (Figure 3). T_0_ plant #1, 3, 4, 7, 8, 11, 12, 14, 16, 17, and 18 had the same 94 bp deletion on the first gRNA region; and plant 8, 13, 16 had deletions of 13 bp and 39 bp on the second gRNA region. Many individual plants had a deletion of the same length in the targeted region. This may indicate an editing hotspot in the target region. Two plants, #6 and 9, had a 355 bp insertion between the two gRNAs, suggesting that larger deletions can be obtained in the region between the two gRNA cutting sites. These results demonstrate 100% editing efficiency using immature embryo calli with *Agrobacterium*-mediated transformation for the *indica* cultivar Ciherang-Sub1.

### 2.5. Optimization of Biolistic Bombardment Procedures

The optimization procedures include testing the optimal calli status, the osmotic medium, setting, and the bombardment system operation. In order to confirm whether the calli are ideal for transformation and validate the biolistic bombardment delivery system, calli were observed and screened under a microscope. The osmotic medium previously reported for bread wheat [42] was tested here for rice calli. To evaluate the transformation, calli were transformed with pPTN-EYFP control vector under 1100 psi helium pressure via Biolistic^®^ PDS-1000/He particle bombardment (BIORAD, Hercules, CA, USA). There are three distances available for the gene gun set up for setting: 3 cm, 6 cm, and 9 cm. We found that 6 cm between calli and the gene gun was the most appropriate distance during the shooting process. The distance of 3 cm was too close, which caused calli to jump out from the medium, whereas the 9 cm distance was too far to allow an efficient transformation. Our results showed that with the 6 cm distance and two shots, the YFP fluorescence could be observed in calli under a fluorescence microscope five days after biolistic bombardment (Figure 1D). This result suggests that the delivery procedure and the callus condition selected are optimal for biolistic bombardment.

### 2.6. Validation of Transgenic Plants Derived from Mature Seeds

After bombardment and recovery from calli that survived under two selection processes and regenerated from SIM and root induction medium (RIM) with hygromycin added, three plants were obtained. After taking out plants from the RIM, plants were transferred to clean water for acclimation. In the meantime, 2 cm leaf pieces from each plant were collected for DNA extraction and validation. The results showed that all the negative controls had no PCR products amplified. In contrast, the positive control and all the three plants had amplified PCR products with the expected sizes (Figure S6).

### 2.7. Phenotyping of T_1_ Transgenic Plants

**Figure 4:**
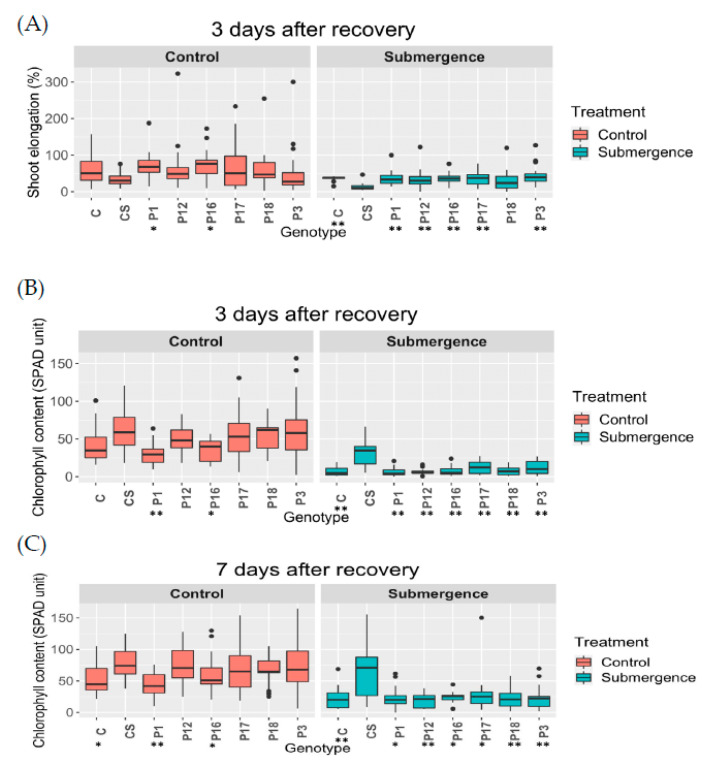
Shoot elongation rate and chlorophyll content of control and submerged plants. (**A**) Shoot elongation rate at three days after desubmergence. Chlorophyll content at three days (**B**) and seven days (**C**) after desubmergence. “C” indicates Ciherang; “CS” indicates Ciherang-Sub1; “P1” indicates plant #1; “P3” indicates plant #3; “P12” indicates plant #12; “P16” indicates plant #16; “P17” indicates plant #17; and “P18” indicates plant #18. “*” indicates *p*-value < 0.05 compared with Ciherang-Sub1; “**” indicates *p*-value < 0.01 compared with Ciherang-Sub1.

**Figure 5:**
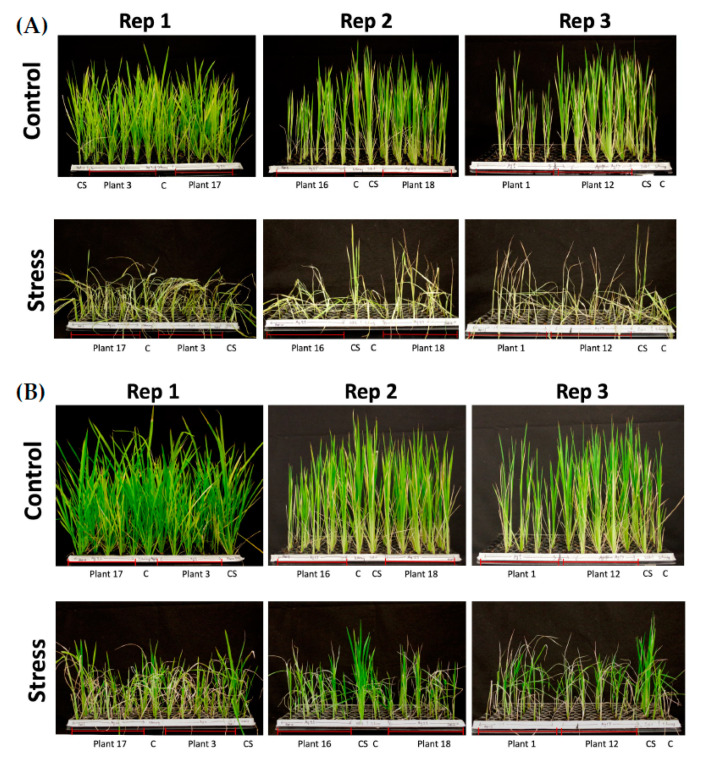
Performance of submerged Ciherang-Sub1, Ciherang, and T_1_ seedlings compared to their corresponding controls. (**A**) Immediately after 14 days of complete submergence, and (**B**) 7 days post-submergence recovery. C, Ciherang; and CS, Ciherang-Sub1.

We used T_1_ seeds derived from the selected six T_0_-edited plants developed from the immature embryo-derived calli via *Agrobacterium*-mediated delivery. Two traits were analyzed in the submergence experiment, shoot elongation rate (%) and chlorophyll content. *SUB1A-1* encodes an ethylene-responsive factor that provides submergence tolerance. [29]. The presence of *SUB1A-1* allows submerged plants to pause shoot elongation during submergence [30]. This phenotype is accompanied with reduced loss of leaf carbohydrates and chlorophyll during submergence. Each tray included 6 Ciherang and 6 Ciherang-Sub1 as negative and positive controls, respectively, along with 30 T_1_ seeds, each from two selected T_0_ plants. The same setup was used for both non-submerged (control) and submerged treatments. There were 3 trays/treatment. In general, the edited plants had a similar performance as Ciherang in both shoot elongation rate and chlorophyll content. Additionally, the control (unsubmerged) groups had better growth than submergence groups (Figure 4 and Figure 5). The results of pairwise comparisons at 3 days after recovery suggest that Ciherang-Sub1 had the lower shoot elongation rate than all other genotypes at *p* < 0.001 except for T_1_ plants derived from plant #18 (*p* = 0.09), and the elongation difference also showed between T_1_ plants derived from plant #18 and those from plant # 3 (*p* < 0.05) (Figure 4A; Table S1). For chlorophyll content, both control (*p* < 0.001) and submergence (*p* < 0.001) groups showed that chlorophyll content increased from 3 days to 7 days after recovery (Figure 4B,C). For the comparison between genotypes, only Ciherang-Sub1 had a higher chlorophyll content than all other genotypes (*p* < 0.05) (Tables S2 and S3). Overall, the phenotypic data analyses of T_1_ transgenic plants indicate that the disruption of *SUB1A* of Ciherang-Sub1 was successful since, in general, the edited plants had Ciherang-like phenotypes.

## 3. Materials and Methods

### 3.1. Plant Materials

The *indica* rice cultivar Ciherang-Sub1 (IR87424-177-173), derived from a cross of a popular rice variety from Indonesia, Ciherang, and a submergence tolerance donor IR64-Sub1 [31], was used.

### 3.2. Construction of the CRISPR-Cas9 Vector

The second exon of *SUB1A* gene was targeted for disruption by editing (Figure S7). Two gRNAs (gRNA1: 5′-CCGGCGAGGAGGCTGTCCATCAC-3′ and gRNA2: 5′-ACGGCCGCTGCCGG ATGCCGTGG-3′) were designed using the design tools CRISPR direct and Cas-OFFinder [43,44]. The gRNAs were inserted into the Golden Gate entry vectors, pYPQ131C (Addgene Plasmid #69284) and pYPQ132C (Addgene Plasmid #69285) [10], that couple an OsU6 promoter to a gRNA, which were then assembled into the Golden Gate recipient vector pYPQ142 (Addgene Plasmid #69294). Next, both pYPQ142 and the Cas9 expression vector (pYPQ150, Addgene Plasmid #69301) were assembled into a binary vector, pMDC32 (Addgene Plasmid #32078), through the LR Clonase II recombination reaction. The final destination vector pMDC32 was transformed into competent *E. coli* (DH5α) cells by heat shock at 42 °C for 30 s. Transformants were selected on kanamycin and confirmed by DNA sequencing. To prepare plasmid DNA for the bombardment, plasmid DNAs were isolated with the QIAprep Spin Miniprep Kit (QIAGEN, Germantown, MD, USA) and stored in a −20 °C freezer.

### 3.3. Immature Embryo Harvest, Tissue Culture, and Transformation

Plants were cultivated in a greenhouse (14 h/10 h (day/night) and 29 °C/26 °C (day/night)) in potting soil in College Station, Texas, and embryos were obtained 10–12 days after pollination. Tissue culture and transformation procedures of immature embryo transformation followed the *Agrobacterium*-mediated transformation procedure for Indica type 4 [15] until the regeneration process. *Agrobacterium tumefaciens* (EHA105)-delivery transformation followed the Hiei and Komari protocol [15], except that the YEB medium was supplemented with kanamycin (50 mg/L). For co-cultivation, immature embryos, with the scutellum face up, were transferred onto NB-As medium. A drop of 5 uL of *Agrobacterium* suspension (O.D. = 1.0 at 660 nm) was added onto each immature embryo. These treated embryos were then incubated for 15 min at 25 °C. Selection was performed on a resting medium (CCMC) with 75 mg/L hygromycin.

### 3.4. Callus Induction from Mature Seeds for Bombardment

The seed coat was removed from healthy mature seeds that were then sterilized with 1.5% (*v*/*v*) sodium hypochlorite and a drop of Tween 20 (100%) for 20 min in a mini rotator (Grant-bio, PTR-25; Grant Instruments, Shepreth, Cambridgeshire, UK) at a speed of 3. Seeds were rinsed in sterile water 7–8 times and air-dried on autoclaved filter paper for 5–10 min. For callus induction, 19–20 seeds were placed onto sterile CIM and incubated at 29 °C in the dark for 14 days. For optimization of CIM, we tested several CIM formulae consisting of 4.4 g/L Murashige and Skoog Basal Medium (MS salts) (Sigma-Aldrich, M404), 0.6 g/L proline, 0.3 g/L casein hydrolysate, 3 mg/L, 3g/L phytagel, pH 5.8), either 30 g/L maltose or 30 g/L sucrose, and several different hormone ratios (2,4-D and 6BA). Petri dishes of 90 mm diameter × 15 mm depth with 40 mL medium were used for all subsequent aseptic tissue culture steps. Calli were dissected from roots, cut into small pieces, and sub-cultured on fresh CIM in Petri dishes (90 mm diameter × 20 mm depth) at 29 °C in the dark for 14–20 days before use. A minimum of 4 h or overnight before the bombardment, calli were transferred to a high-osmotic medium (OSM) [42] and incubated at 23 °C in the dark. OSM was prepared using 4.4 g/L MS salts, 5 mg/L 2,4-D, 72.87 g/L mannitol, pH 5.8, and 3.2 g phytagel.

### 3.5. Plasmid Preparation and Biolistic Bombardment

The biolistic transformation of calli was performed using the PDS-1000/He particle bombardment system with 1.0 µm gold particles at a helium pressure of 1100 psi with a target distance of 6 cm. The bombardment procedures followed the Biolistic^®^ PDS-1000/He Particle Delivery System user manual [45]. Before coating gold particles with DNA, 30 mg of 1.0 µm gold particles were sterilized with 70% (*v*/*v*) EtOH and resuspended with 50% (*v*/*v*) glycerol. These were stored at −20 °C for up to 2 weeks. The following steps were performed while vortexing continually: A 50 μL aliquot of gold particles was combined with 5 μg of DNA to a total volume of 55 μL using 2.5 M cold CaCl2, after which 20 μL of 0.1 M spermidine was added. The mix was vortexed for 3 min, the gold particles pelleted in a microcentrifuge, and the supernatant discarded. The DNA-coated gold particles were resterilized in 100 mL 100% (*v*/*v*) EtOH, pelleted, and resuspended in 48 μL 100% EtOH. These gold particles were vortexed at low speed, and 7 μL was applied onto the dry macrocarriers. Two 1100 psi shots were applied to each plate of tissue. After bombardment, calli were incubated in OSM overnight in the dark at 23 °C. The next day, calli were transferred onto fresh CIM and cultured at 29 °C in the dark for 7–10 days until transfer to selection medium (MSM; CIM containing 50 mg/L hygromycin) and cultured at 27 °C in the dark for 12 days. Brown or black calli were removed, and yellow or cream-colored calli were transferred to fresh MSM for a second selection period of 10–12 days. Sections of small new light-colored calli were transferred onto regeneration medium in Petri dishes (90 mm diameter × 20 mm depth). A third selection was performed sometimes.

### 3.6. Callus Regeneration for Both Immature Embryos and Mature Seeds

The same shoot regeneration medium was used for calli-induced mature seeds and immature embryos. Shoot induction medium (SIM) was modified from two studies [15,19]. To identify the best growth condition for regeneration medium for Ciherang-Sub1, after selection on hygromycin, calli were transferred onto SIM with 30 mg/L hygromycin (4.4 g/L MS salts, 30 g/L maltose, with two different ratios of kinetin and 1-Naphthaleneacetic acid (NAA), and two different concentration of agarose g/L agarose, pH 5.8) and cultured at 29 °C with a day/night period 14/10 h (light intensity was 405 µmol/m^2^/s) in Petri dishes (90 mm diameter × 15 mm depth) or Magenta GA-7 vessels (100 mL medium). In the first stage of shooting, calli were transferred onto R1 Petri dishes for 14 days until the calli turned green and shoot differentiation was visible. Calli with green shoots were transferred to fresh R1 in a Magenta vessel for 7–10 days until the plants were 5 cm or taller and three or more tillers.

Plantlets were then transferred to the root induction medium (RIM; containing 2.2 g/L MS salts, 30 g/L sucrose, 3 g/L phytagel, pH 5.8, containing 30 mg/L hygromycin) in Magenta vessels (100 mL) and returned to the growth chamber until the leaves reached the lid (7–10 days). The lid was then removed and replaced by an empty, inverted Magenta GA-7 vessel for another 5–7 days of culture. Plants were removed from the medium, roots were cleaned well with tap water, removed of all the medium residues, and plants were placed into a Magenta GA-7 vessel filled with fresh tap water in a well-ventilated area at 23–25 °C, out of direct sunlight for 7–10 days of acclimation. Once new roots formed, plants were finally transferred to pots containing a 1:1 mixture of Metro Mix 820 Potting soil and Turface Athletics MVP and moved to the greenhouse.

### 3.7. Validation of Transgenic Plants

DNA was extracted from the young leaves of each regenerated plant using a modified CTAB method [46]. Plants were genotyped using pYPQ150 Cas9 specific primers (Forward: 5′-TCGGTGCTCTTCTTTTCGAT-3′; Reverse: 5′-TTCCGAGATTGGGTGTCTC-3′) and/or *hygromycin B phosphotransferase* gene (HPT) primers (Forward: 5′-CGCATAA-CAGCGGTCATTGACTGGAGC-3′; Reverse: 5′-GCTGGGGCGTCGGTTTCCACTATCC-G-3′). Plants having positive bands for these markers were further examined by mutations within the target region of *SUB1A-1* using gene-specific primers (Forward: 5′-CGGTAGATGCCGAGAAGTGT-3′; Reverse: 5′- GTGGACTATGCGATGTGTGG-3′) and TA cloning-based Sanger sequencing. To confirm the edited region, three colonies from each target were sequenced, totaling six colonies/sample. The expected product size of the HPT marker was 375 bp. DNA of transgenic samples, positive control, and negative controls were diluted to 100–150 ng for the PCR reaction with an annealing temperature of 55 °C.

### 3.8. Calculation of Regeneration and Transformation Efficiency

The regeneration efficiency was calculated as the number of regenerated calli among the total calli on the regeneration medium. To test the optimal regeneration medium, the regeneration medium was tested using untransformed healthy calli without antibiotics. The editing efficiency was calculated as the number of edited plants among the total number of transgenic plants (i.e., regenerated plants with both selective markers positive).

### 3.9. Submergence Experiment and Phenotyping of T_1_ Plants

T_1_ seeds derived from mutant T_0_ plants were selected for phenotyping. These were germinated in a 6 × 12 holes plastic tray and grown in a growth chamber with 12/12 h for day/night length and 29 °C/26 °C for day/night, with a light intensity of 600–1000 PAR. On day 14 after germination, seedlings were completely submerged in 100 cm of water in a plastic tank for 16 days. Following de-submergence, trays were returned to the greenhouse bench. Control plants were maintained in the same greenhouse environment. Shoot elongation was measured from the base of the shoot to the tip of the longest leaf. Chlorophyll content was measured in the third leaf in control and treated plants using an Apogee MC-100 Chlorophyll Meter (Apogee Instruments, Inc., Logan, UT, USA)

### 3.10. Phenotypic Data Analysis

First, the normality of phenotypic data was examined by the Shapiro–Wilk test. The results indicated that both shoot elongation rate and chlorophyll content violated the normal distribution; therefore, a non-parametric Kruskal–Wallis test was used to examine the effects. A Wilcoxon rank-sum test was then used for multiple comparisons.

## 4. Conclusions

The results of our study show that it is possible to obtain high regeneration and high editing efficiency in Ciherang-Sub1 using CRISPR-Cas9 with immature embryos as explants through *Agrobacterium*-mediated transformation. In addition, we demonstrated a potential biolistic bombardment transformation protocol using mature *indica* seeds as explants for CRISPR-Cas9 gene editing. With all the necessary media, including callus induction, osmotic, selection, and regeneration media, the mature seed protocol allowed us to obtain transgenic plants within 80 days. A high regeneration rate of 95% was obtained from *indica* rice cultivars Ciherang-Sub1 using the optimized SIM, and there is a potential use of this medium for other *indica* varieties as well. This regeneration medium can be used in both calli derived from mature seeds and immature embryos and can be adapted to both *Agrobacterium*-mediated and biolistic bombardment transformation procedures. This study will enable further investigation of additional submergence tolerance gene(s) in the background of Ciherang-Sub1 after removing the large effect of the major *SUB1A-1* gene. Further, this also provides an opportunity for future efforts to further improve Ciherang-Sub1 and potentially other *indica* rice varieties via gene-editing and genetic engineering.

## Data Availability

Not applicable.

## Supplementary Materials

The following are available online at https://www.mdpi.com/article/10.3390/ijms22136989/s1.

## Abbreviations

2:4-D 6BA CIM CRISPR-Cas9	2,4-Dichlorophenoxyacetic acid 6-Benzylaminopurine Callus induction medium Clustered regularly interspaced short palindromic repeats-Cas9
gRNA	Guide RNA
HR	Homologous recombination
MS NAA NEHJ RIM SIM *SUB1*	Murashige and Skoog 1-Naphthaleneacetic acid Non-homologous end-joining Root induction medium Shoot induction medium *SUBMERGENCE 1*
WT	Wild type

## Appendix A

**Table A1:** Comparison of callus induction media with different cytokinin to auxin ratios.

Components	Formula (1) [19]	Formula (2) [22]	Formula (3)	Formula (4)	Formula (5)
2,4-D (2,4-Dichlorophenoxyacetic acid)	3 mg/L	2.5 mg/L	3 mg/L	3 mg/L	3 mg/L
6BA (6-Benzylaminopurine)	0.25 mg/L	0.15 mg/L	0.2 mg/L	0.2 mg/L	0.3 mg/L
Phytagel	3 g/L	4 g/L	3 g/L	4 g/L	3 g/L
Agarose	-	2 g/L	-	-	-

**Table A2:** Comparison of regeneration media with different cytokinin to auxin ratios.

Components	Formula (1) [19]	Formula (2)	Formula (3)
Kinetin	2 mg/L	3 mg/L	3 mg/L
NAA (1-Naphthaleneacetic acid)	0.2 mg/L	0.1 mg/L	0.1 mg/L
Agarose	8 g/L	8 g/L	7 g/L
