# Supplementary material for: Improved Transformation and Regeneration of Indica Rice: Disruption of SUB1A as a Test Case via CRISPR-Cas9
Source: Int J Mol Sci. 2021 Jun 29;22(13):6989. doi: 10.3390/ijms22136989 (PMC8269137; doi:10.3390/ijms22136989)
Supplement: Supplementary file 1 [file ijms-22-06989-s001.zip › ijms-1276017-supplementary.pdf]

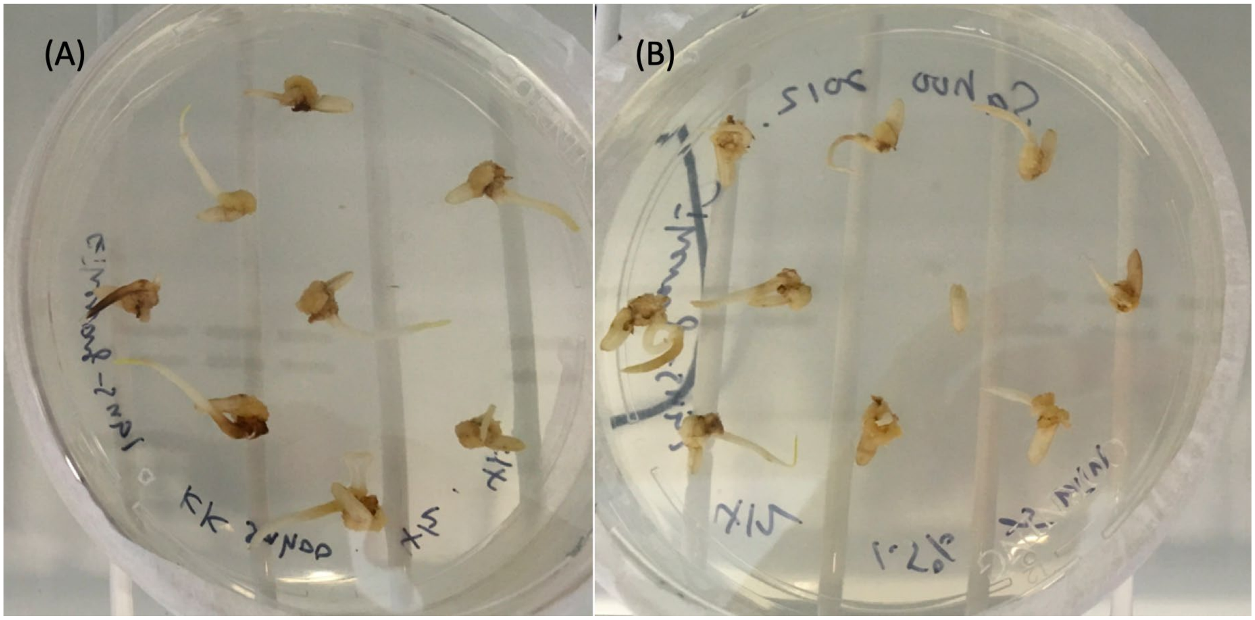

Figure S1. Callus induced from mature seeds after 10 days culture in different callus induction media. (A) Formula 1 [19] (auxin:cytokinin = 3:0.25) and (B) Formula 2 [31] (auxin:cytokinin = 2.5:0.15).

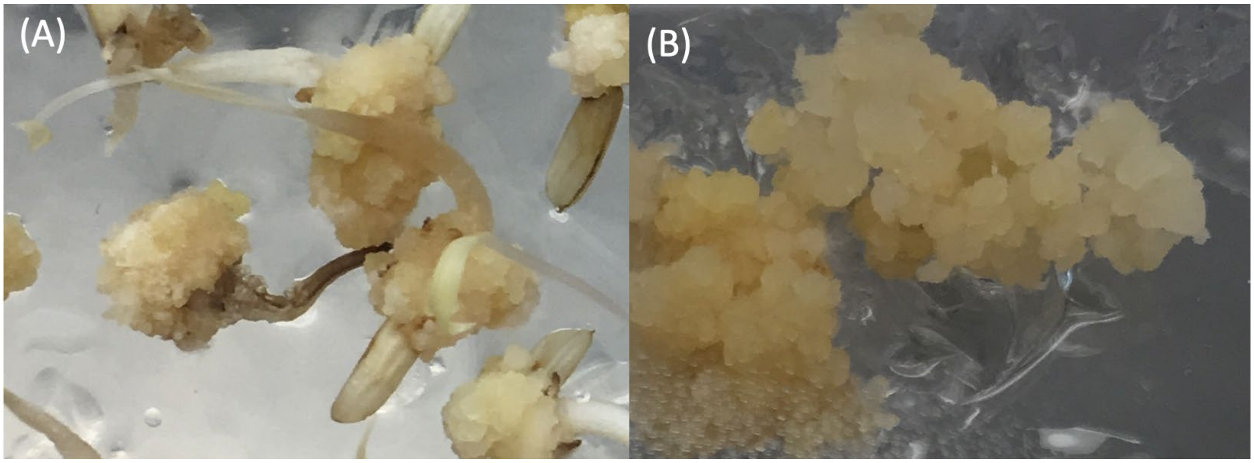

Figure S2. Calli from mature seeds after culture in callus induction medium (formula 5) for 14 d (A) and 16 days (B).

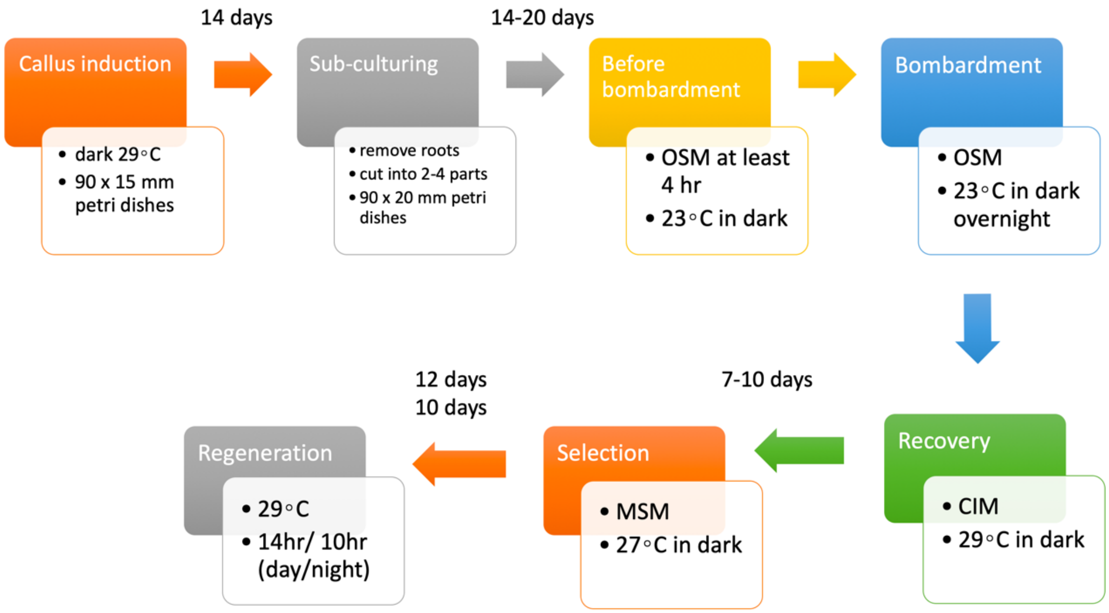

Figure S3. Biolistic bombardment workflow for Ciherang-Sub1 using mature seeds as explants.

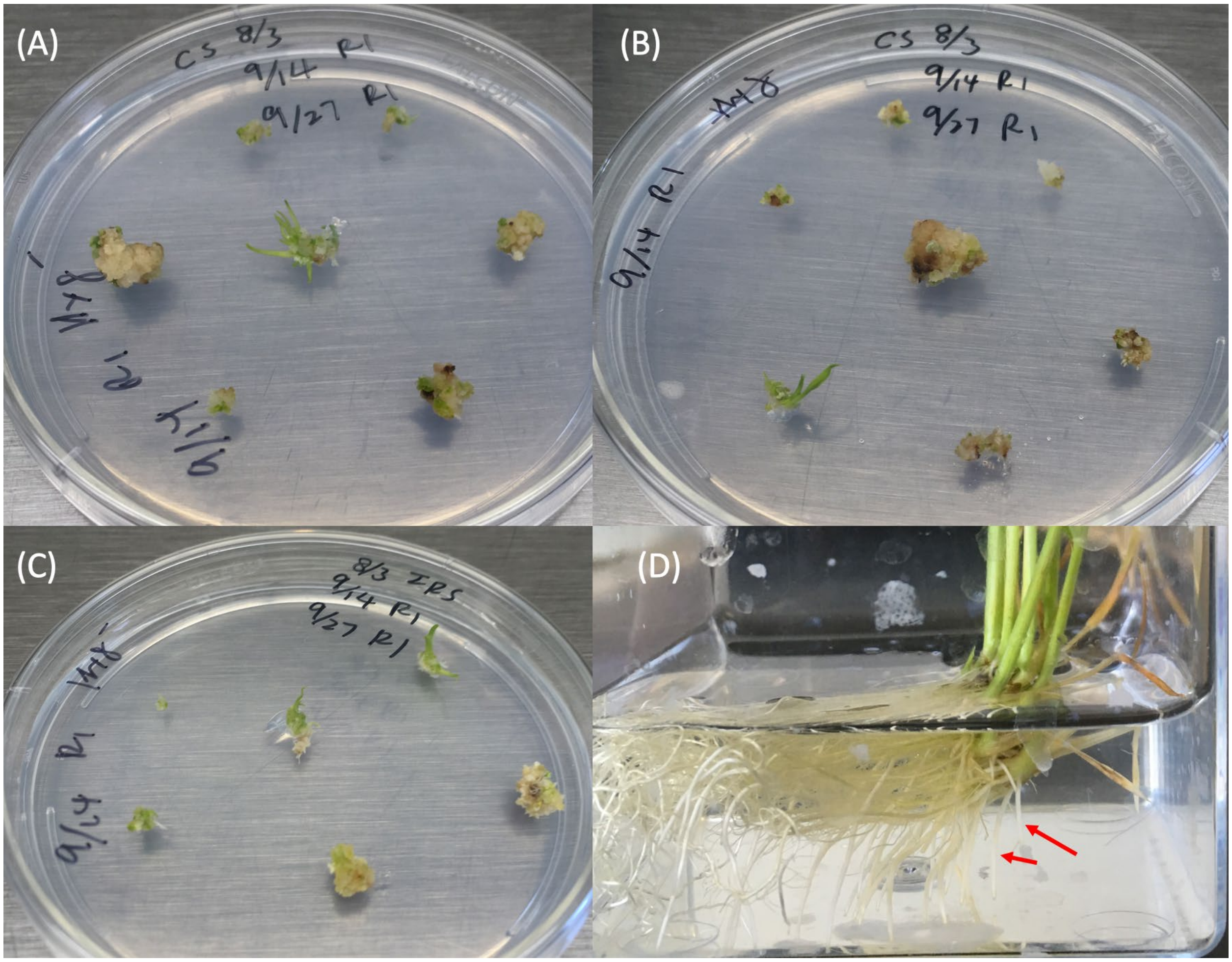

Figure S4. Untransformed calli derived from mature seeds, after 13 days of culture on optimal regeneration medium (formula 3) without antibiotic added. (A) and (B) Ciherang-Sub1. (C) IR64-Sub1. (D) Plant acclimation in fresh tap water. Red arrows indicate the newly formed roots.

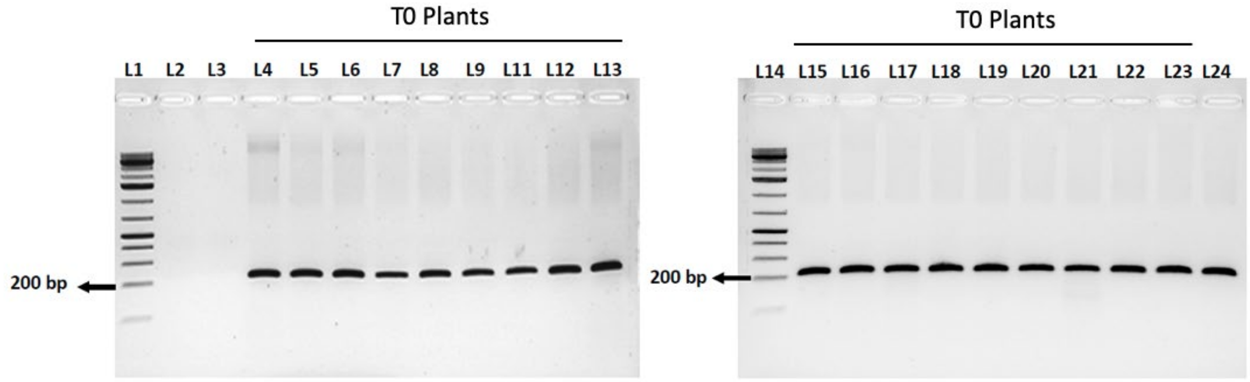

Figure S5. Validation of Cas9 gene presence in transgenic plants (T_0) derived from immature embryos with Cas9-specific primers: L1 and L14: 1kb+ ladder; L2: water control; L3: wild type Ciherang-Sub1; L4-L23: T0 plants (Plant no.: Plant 1, Plant 2, Plant 3, Plant 4, Plant 5, Plant 6, Plant 7, Plant 8, Plant 9, Plant 10, Plant 11, Plant 12, Plant 13, Plant 14, Plant 15, Plant 16, Plant 17, and Plant 18) and L24: positive control

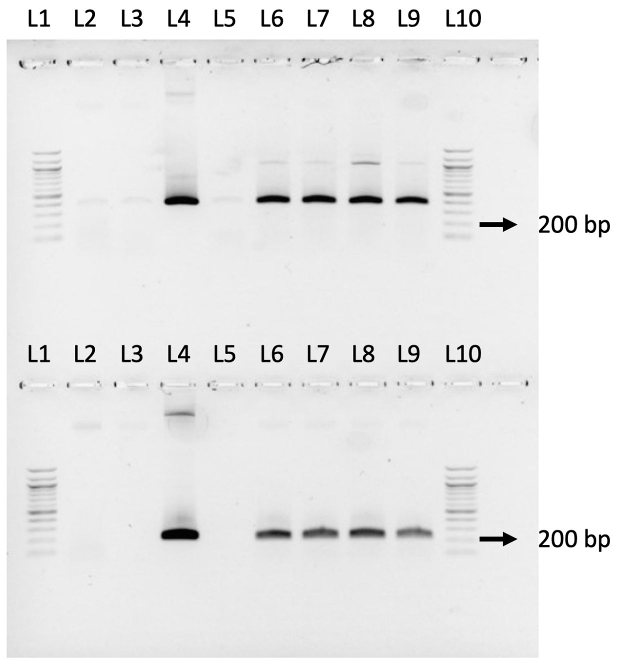

Figure S6. Validation of transgenic plants (T_0) derived from mature seeds with HPT primers and Cas9 specific primers. Upper panel HPT primers; lower panel Cas9 specific primers. L1 and L10: 1kb+ ladder; L2: Ciherang; L3: wild type Ciherang-Sub1; L4: positive control; L5: water control; and L6-L9: transformed plants (Plant no.: Bombardment 1 leaf 1, Bombardment 1 leaf 2, Bombardment 2, and Bombardment 3).

```
Query  1    GGTGTTGAGGTCACCAGGTGAAAATGATGCAGGCCGGGGCGCCGCCGCCACCATGTCCAT  60
            ||||||||||||||||||||||||||||||||||||||||||||||||||||||||||||
Sbjct  675  GGTGTTGAGGTCACCAGGTGAAAATGATGCAGGCCGGGGCGCCGCCGCCACCATGTCCAT  616
Query  61   GCCGCTGGACCCCGTGACCGAGGAGGCCGAGCCGGCGGTGGCTGAGAAGCCTCGCCGGCG  120
            ||||||||||||||||||||||||||||||||||||||||||||||||||||||||||||
Sbjct  615  GCCGCTGGACCCCGTGACCGAGGAGGCCGAGCCGGCGGTGGCTGAGAAGCCTCGCCGGCG  556
Query  121  CCGGCCGAGGCGGAGCTACGAGTACCACGGCATCCGGCAGCGGCCGTGGGGGCGGTGGTC  180
            ||||||||||||||||||||||||||||||||||||||||||||||||||||||||||||
Sbjct  555  CCGGCCGAGGCGGAGCTACGAGTACCACGGCATCCGGCAGCGGCCGTGGGGGCGGTGGTC  496
Query  181  GTCGGAGATCCGCGACCCCGTCAAGGGCGTCCGCCTCTGGCTCGGCACCTTCGACACCGC  240
            ||||||||||||||||||||||||||||||||||||||||||||||||||||||||||||
Sbjct  495  GTCGGAGATCCGCGACCCCGTCAAGGGCGTCCGCCTCTGGCTCGGCACCTTCGACACCGC  436
Query  241  CGTCGAAGCCGCGCTCGCCTACGACGCCGAGGCCCGCCGCATCCACGGCTGGAAAGCCCG  300
            ||||||||||||||||||||||||||||||||||||||||||||||||||||||||||||
Sbjct  435  CGTCGAAGCCGCGCTCGCCTACGACGCCGAGGCCCGCCGCATCCACGGCTGGAAAGCCCG  376
Query  301  GACAAACTTCCCACCCGCCGATCTTTCTTCGCCGCCGCCGCCGTCGCAGCCGCTCTGCTT  360
            ||||||||||||||||||||||||||||||||||||||||||||||||||||||||||||
Sbjct  375  GACAAACTTCCCACCCGCCGATCTTTCTTCGCCGCCGCCGCCGTCGCAGCCGCTCTGCTT  316
Query  361  CTTGCTCAACGACAACGGCCTCATCACAATCGGAGAAgcgccgaccgacgacgccgcgtc  420
            ||||||||||||||||||||||||||||||||||||||||||||||||||||||||||||
Sbjct  315  CTTGCTCAACGACAACGGCCTCATCACAATCGGAGAAGCGCCGACCGACGACGCCGCGTC  256
Query  421  gacgtcgacgtcgacgacggaggcgtccggcgacgcgcgcATACAACTGGAGTGCTGCTC  480
            ||||||||||||||||||||||||||||||||||||||||||||||||||||||||||||
Sbjct  255  GACGTCGACGTCGACGACGGAGGCGTCCGGCGACGCGCGCATACAACTGGAGTGCTGCTC  196
Query  481  GGACGACGTGATGGACAGCCTCCTCGCCGGCTACGACGTGGCCAGCGGCGACGACATATG  540
            ||||||||||||||||||||||||||||||||||||||||||||||||||||||||||||
Sbjct  195  GGACGACGTGATGGACAGCCTCCTCGCCGGCTACGACGTGGCCAGCGGCGACGACATATG  136
Query  541  GACATGGACATCTGGAGCCTCCTCCACCTCTGTTAACCAAGAGATCAAGACCCCATCGAT  600
            ||||||||||||||||||||||||||||||||||||||||||||||||||||||||||||
Sbjct  135  GACATGGACATCTGGAGCCTCCTCCACCTCTGTTAACCAAGAGATCAAGACCCCATCGAT  76
Query  601  C  601
            |
Sbjct  75   C  75
```

Figure S7. *SUB1A-1* sequence alignment between Ciherang-Sub1 and the second exon of *SUB1A-1* (NCBI Accession DQ011598.1) with the two gRNAs marked in yellow.

Table S1. Multiple comparisons for the shoot elongation rate among T_1 plants, Ciherang, and Ciherang-Sub1.

	Ciherang	Ciherang-Sub1	Plant 1	Plant 12	Plant 16	Plant 17	Plant 18
Ciherang-Sub1	0.00097	-	-	-	-	-	-
Plant 1	0.94808	0.00058	-	-	-	-	-
Plant 12	0.59060	0.00226	0.93535	-	-	-	-
Plant 16	0.94808	0.00057	1.00000	0.64593	-	-	-
Plant 17	0.93535	0.00057	1.00000	0.85778	0.94808	-	-
Plant 18	0.25915	0.09419[a]	0.25915	0.49611	0.23746	0.23746	-
Plant 3	0.59060	0.00015	0.61329	0.30516	0.59060	0.59060	0.04790[b]

[a]No significant difference between Ciherang-Sub1 plants and T_1 plants derived from plant # 18.

[b]There was significant difference between T_1 plants derived from plant #18 and T_1 plants derived from plant #3.

Table S2. Multiple comparisons for the leaf chlorophyll content after 3 days of recovery among T_1 plants, Ciherang, and Ciherang-Sub1.

	Ciherang	Ciherang-Sub1	Plant 1	Plant 12	Plant 16	Plant 17	Plant 18
Ciherang-Sub1	0.00097	-	-	-	-	-	-
Plant 1	0.89779	0.00018	-	-	-	-	-
Plant 12	0.69488	0.00018	0.77070	-	-	-	-
Plant 16	0.73488	0.00026	0.69488	0.88511	-	-	-
Plant 17	0.19869	0.00121	0.15207	0.19702	0.11085	-	-
Plant1 8	0.67814	0.00013	0.68105	0.67814	0.89779	0.22085	-
Plant 3	0.19869	0.00121	0.16266	0.22085	0.25724	0.93660	0.22085

Table S3. Multiple comparisons for the leaf chlorophyll content after 7 days of recovery among T_1 plants, Ciherang, and Ciherang-Sub1.

	Ciherang	Ciherang-Sub1	Plant 1	Plant 12	Plant 16	Plant 17	Plant 18
Ciherang-Sub1	0.0168	-	-	-	-	-	-
Plant 1	0.9484	0.0168	-	-	-	-	-
Plant 12	0.9335	0.0085	0.8904	-	-	-	-
Plant 16	0.8904	0.0214	0.6904	0.6740	-	-	-
Plant 17	0.8628	0.0214	0.8904	0.4874	0.9900	-	-
Plant 18	0.9803	0.0085	0.9900	0.8628	0.8904	0.8628	-
Plant 3	0.9803	0.0085	0.9484	0.9335	0.6159	0.6159	0.9335
